# Longitudinal Quality-of-Life Trajectories Following Laparoscopic Distal Gastrectomy: A Comparison Between Billroth I and II Reconstruction Using the KOQUSS-40 Questionnaire

**DOI:** 10.3390/jcm15103738

**Published:** 2026-05-13

**Authors:** Jae Yeong Yang, Ji Yeon Park, Oh Kyoung Kwon, Ki Bum Park

**Affiliations:** 1 Department of Gastrointestinal Surgery, Kyungpook National University Chilgok Hospital, Daegu 41404, Republic of Korea; stomachyang22@knuh.kr (J.Y.Y.); jybark99@knu.ac.kr (J.Y.P.); okugisurg@knu.ac.kr (O.K.K.); 2 Department of Surgery, School of Medicine, Kyungpook National University, Daegu 41566, Republic of Korea

**Keywords:** gastric cancer, distal gastrectomy, Billroth I, Billroth II, quality of life, KOQUSS-40

## Abstract

**Background/Objectives:** Although Billroth I (BI) and Billroth II (BII) are standard reconstructions after distal gastrectomy, evidence on their longitudinal quality-of-life (QoL) trajectories remains inconclusive. This study compared postoperative QoL patterns between BI and BII groups using a validated gastric cancer-specific instrument. **Methods:** We analyzed 234 patients (BI *n* = 51, BII *n* = 183) who underwent laparoscopic distal gastrectomy. QoL was assessed using the KOQUSS-40 questionnaire at 1, 3, 6, 9, and 12 months postoperatively. To address selection bias, inverse probability of treatment weighting (IPTW) was applied alongside linear mixed-effects models to evaluate group effects and group × time interactions. **Results:** A significant main effect of group was observed (*p* = 0.032), with the BII group maintaining higher total scores. These findings remained consistent after IPTW adjustment. While the overall group × time interaction was not significant (*p* = 0.846), indicating parallel recovery trajectories, significant interactions were identified in specific domains, including dumping syndrome and constipation. In these domains, the BI group showed gradual improvement, whereas the BII group exhibited relatively stable scores. **Conclusions:** While both reconstruction methods demonstrated comparable overall QoL trajectories with no significant group × time interaction, the KOQUSS-40 revealed exploratory differences in specific symptom domains, notably dumping-related symptoms and constipation. These findings provide clinically meaningful insights for postoperative patient counseling, enabling tailored expectations during the first year after distal gastrectomy. However, these domain-specific results should be regarded as hypothesis generating and interpreted with caution.

## 1. Introduction

Gastric cancer remains one of the most prevalent malignancies worldwide, with particularly high incidence rates in East Asian countries, including South Korea [1]. The widespread implementation of national cancer screening programs has led to a significant increase in the diagnosis of early gastric cancer (EGC) in recent years [2,3]. Given the excellent prognosis of EGC, with five-year survival rates exceeding 90% following surgical resection, preserving and improving postoperative quality of life (QoL) has become a crucial long-term therapeutic goal alongside oncologic outcomes [2].

Distal gastrectomy, the standard surgical treatment for EGC, has evolved considerably with the advent of minimally invasive techniques [3,4]. Totally laparoscopic distal gastrectomy (TLDG), in which all procedures are performed intracorporeally, is now widely adopted because it reduces postoperative pain, shortens recovery time, and offers superior cosmetic outcomes compared with open or laparoscopic-assisted approaches. Among reconstruction methods following distal gastrectomy, Billroth I (BI) and Billroth II (BII) anastomoses are the most commonly performed [3]. BI anastomosis restores continuity between the remnant stomach and the duodenum, preserving a more physiologic food pathway [5]. However, this technique may be associated with increased anastomotic tension. In contrast, BII anastomosis connects the remnant stomach to the jejunum and offers greater technical ease and lower anastomotic tension, though concerns regarding bile reflux remain. Despite the widespread use of both techniques, their impact on postoperative QoL remains debated [6].

Although several studies have compared QoL outcomes between BI and BII reconstructions, most have utilized Western-based assessment tools such as the European Organization for Research and Treatment of Cancer Quality of Life Questionnaire Core 30 (EORTC QLQ-C30) and the gastric cancer-specific module (QLQ-STO22) [7,8]. However, these instruments may not be specifically tailored to patients in Korea who have undergone gastrectomy. To address these limitations of the EORTC, the Korean Quality of Life in Stomach Cancer Patients Study Group (KOQUSS)-40 questionnaire was developed to capture nuanced, surgery-specific symptoms and lifestyle adaptations [9]. This study aimed to precisely compare the distinct symptomatic profiles and subtle recovery trajectories between BI and BII reconstruction using the KOQUSS-40 questionnaire.

Given the limited application of this instrument in Korean patients undergoing TLDG, we evaluated short-term postoperative QoL after BI versus BII reconstruction, with particular emphasis on symptom trajectories during the first postoperative year.

## 2. Materials and Methods

### 2.1. Patients

This study was approved by the Institutional Review Board (IRB) of Kyungpook National University Chilgok Hospital (IRB no. 2025-02-003-002). Initially, 279 consecutive patients with histologically confirmed gastric cancer who underwent a TLDG with either BI or BII reconstruction between August 2022 and August 2023 were identified at our institution.

The exclusion criteria were as follows:

1. A history of prior cancer surgery involving other organs (*n* = 11).
2. Ongoing treatment for other significant diseases that could affect quality of life (*n* = 9).
3. A confirmed diagnosis of synchronous cancer in another organ, tumor recurrence, or mortality within the first postoperative year (*n* = 12).
4. Loss to follow-up before the 1-year postoperative assessment (*n* = 13).

After applying these criteria, 234 patients were included in the final analysis. Patients were divided into two groups according to the reconstruction method: the BI anastomosis group (*n* = 51) and the BII anastomosis group (*n* = 183).

### 2.2. Surgical Procedure

All TLDG procedures were performed by experienced gastrointestinal surgeons according to the Japanese and Korean Gastric Cancer Treatment Guidelines [10,11]. Following standard distal gastric resection with D1+ or D2 lymph node dissection, gastrointestinal reconstruction was performed. The choice between BI and BII anastomosis was at the discretion of the operating surgeon and was based on factors such as tumor location, remnant stomach size, and duodenal stump tension.

For BI reconstruction, an intracorporeal delta-shaped gastroduodenostomy was performed using linear staplers. Small entry holes were created in the remnant stomach and duodenum, after which the posterior walls of both were approximated to construct a side-to-side anastomosis using a 45-mm linear stapler. The common entry hole was subsequently closed with another application of a 60-mm linear stapler. Both the anastomosis and the closure of the entry hole were performed through a 12-mm port in the left flank area [12,13]. BII reconstruction was performed using an antecolic, side-to-side gastrojejunostomy in either an isoperistaltic or antiperistaltic fashion. The anastomosis was created 25 cm distal to the ligament of Treitz using a 60-mm linear stapler, followed by closure of the common entry hole with an additional 60-mm linear stapler [13,14].

### 2.3. QoL Assessment

Postoperative QoL was the primary outcome measure and was assessed using the validated KOQUSS-40 questionnaire [9]. The KOQUSS-40 is a 40-item, symptom-focused instrument specifically designed to evaluate the QoL of patients who have undergone gastrectomy. It covers eight distinct domains: indigestion, dysphagia, reflux, dumping syndrome, bowel habit changes, constipation, psychological factors, and worry about cancer. Scoring was performed according to the method described in a previous KOQUSS group study, with higher scores indicating better QoL [9]. QoL was evaluated at five longitudinal time points. The first assessment, designated as ‘postoperative 1 month,’ was conducted during the first outpatient clinic visit after discharge (typically 2–4 weeks postoperatively). Subsequent evaluations were performed at 3, 6, 9, and 12 months after surgery. patients completed the questionnaire independently upon arrival at the outpatient clinic, prior to physician consultation. Questionnaire data collected between August 2022, and May 2024 were analyzed to compare the 1-year QoL outcomes between the BI and BII groups.

### 2.4. Statistical Analysis

Categorical variables were summarized using frequencies and percentages, and continuous variables as mean ± standard deviation. Group differences were assessed using the chi-square test or Fisher’s exact test for categorical data and the independent *t*-test or Mann–Whitney U test for continuous data, as appropriate. Normality of data distribution was assessed using the Shapiro–Wilk test.

Generalized linear mixed-effects models (GLMMs) were fitted to the repeated-measures data to evaluate longitudinal QoL outcomes. In each model, fixed effects included the group effect (to assess the overall mean difference between the BI and BII groups across all time points), the time effect (to evaluate temporal changes common to both groups), and the group × time interaction (to assess differences in recovery trajectories between the two groups). A random intercept for each participant was included to account for between-subject variability, and an unstructured covariance matrix was specified to model within-subject correlations over time.

**Figure 1:**
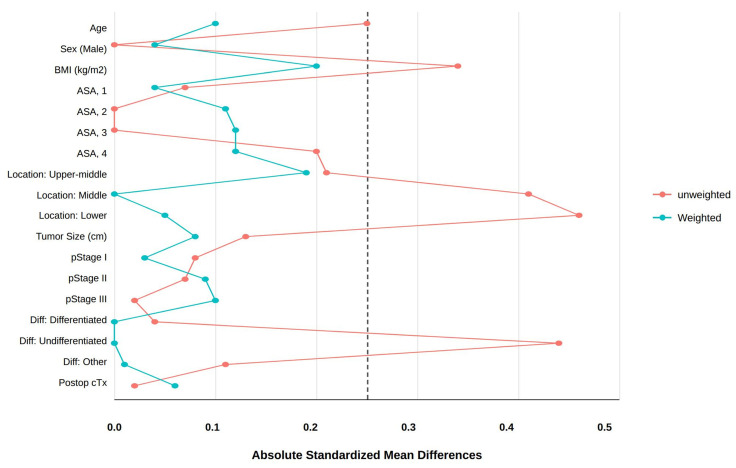
Absolute standardized difference (ASD) plot before and after inverse probability of treatment weighting (IPTW). ASD < 0.25 implies good balance between the two groups. The dashed line indicates an ASD of 0.25. Abbreviations: BI, Billroth I anastomosis; BII, Billroth II anastomosis; BMI: body mass index; ASA: American Society of Anesthesiologists; pStage: pathologic stage; Postop cTx: postoperative chemotherapy.

To further address potential confounding and selection bias due to the non-randomized study design, inverse probability of treatment weighting (IPTW) based on propensity scores (PS) was applied as a sensitivity analysis. PS were estimated using a multivariable logistic regression model including baseline clinicopathologic variables (age, sex, body mass index (BMI), American Society of Anesthesiologists (ASA) physical status, tumor location, pathological stage (pStage), and histologic differentiation). IPTW was calculated as 1/PS for patients in the BII group and 1/(1 − PS) for those in the BI group. To evaluate the balance of baseline covariates before and after IPTW, both absolute standardized differences (ASDs) and a visual assessment using a Love plot were employed. Following the guidelines of Rubin [15], an ASD of less than 0.25 was used as the threshold to indicate a negligible imbalance between the groups. Visual inspection of the Love plot (Figure 1) confirmed that all weighted covariates fell within this predefined threshold, ensuring that the two cohorts were sufficiently comparable. The IPTW-adjusted results were compared with the primary GLMM findings to ensure the robustness of the longitudinal QoL trajectories.

In addition, to control for residual confounding by indication, clinicopathologic variables showing significant baseline differences (e.g., tumor location and histologic differentiation) were further included as fixed-effect covariates in the models. These variables were adjusted for as potential confounders, although their independent impact on longitudinal functional QoL was considered limited relative to the physiological effects of reconstruction. Outcome values were plotted over time, with time on the horizontal axis and outcome measures on the vertical axis, and data points connected by lines. All statistical analyses were performed using SPSS version 29.0 (IBM Corp., Armonk, NY, USA). A *p*-value less than 0.05 was considered statistically significant. As this study was exploratory in nature, no formal adjustment for multiple comparisons was applied to the analysis of individual QoL domains. Consequently, these domain-specific findings should be interpreted as hypothesis generating rather than definitive, and results for specific symptoms should be viewed with caution.

## 3. Results

Baseline demographic characteristics were generally comparable between the two groups (Table 1). There were no statistically significant differences in mean age (63.06 ± 10.49 for BI vs. 65.63 ± 10.08 for BII; *p* = 0.149), sex distribution (*p* = 0.980), BMI (23.29 ± 3.20 for BI vs. 24.45 ± 3.55 for BII; *p* = 0.059), and ASA physical status classification (*p* = 0.413).

Analysis of clinicopathologic features revealed statistically significant differences in tumor location and differentiation. The BI group had a significantly higher proportion of tumors located in the lower third of the stomach compared to the BII group. Conversely, the BII group had a higher incidence of tumors in the middle third of the stomach (lower third: 94.1% vs. 78.6%, respectively, middle third: 5.9% vs. 19.1%, respectively; *p* = 0.033). Regarding tumor histology, the BI group had a significantly higher rate of undifferentiated tumors than the BII group (62.7% vs. 41.5%), while the BII group had a higher rate of differentiated tumors (54.6% vs. 35.3%; *p* = 0.024). Other clinicopathologic characteristics, including mean tumor size (2.38 ± 1.24 cm for BI vs. 2.54 ± 1.17 cm for BII; *p* = 0.246), pathologic T stage (*p* = 0.551), pathologic N stage (*p* = 0.212), overall pStage (*p* = 0.925), and the proportion of patients receiving postoperative chemotherapy (13.7% for BI vs. 13.1% for BII; *p* = 0.909), showed no statistically significant differences between the two groups. 

**Figure 2:**
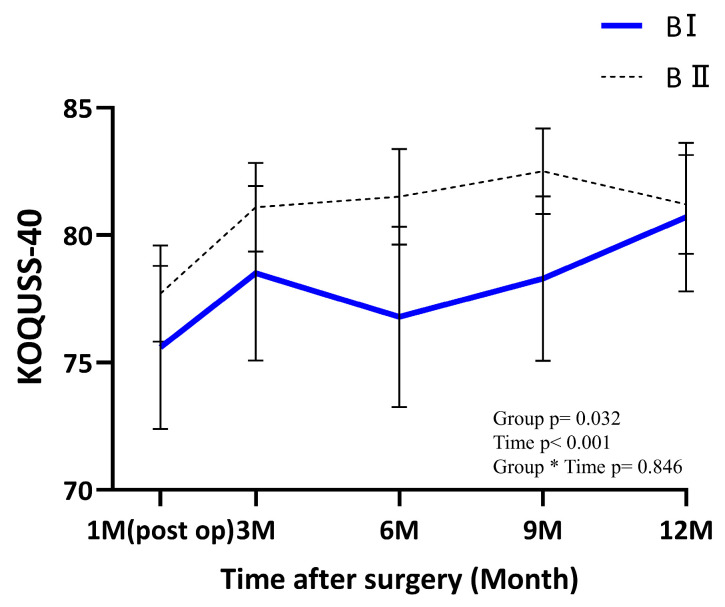
KOQUSS-40 total score according to anastomosis type. Lines represent mean values, and shaded areas indicate 95% confidence intervals. Time points: Postop 1 month (first outpatient visit), 3 months, 6 months, 9 months, and 12 months. The asterisk (*) represents the interaction effect between the reconstruction group and the postoperative time points. Abbreviations: KOQUSS, Korean Quality of Life in Stomach Cancer Patient Study; BI, Billroth I anastomosis; BII, Billroth II anastomosis.

**Figure 3:**
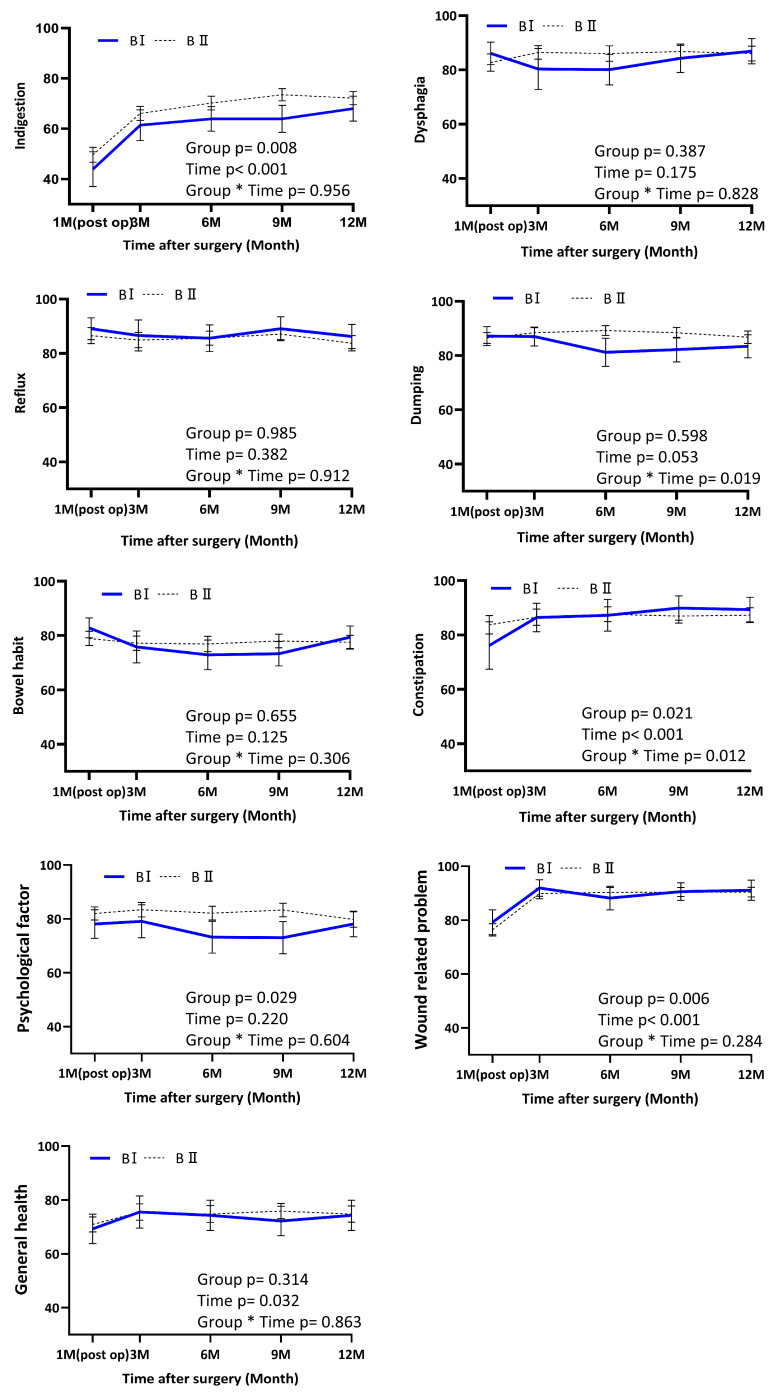
Score of the KOQUSS-40 domain according to the anastomosis type. Lines represent mean values, and shaded areas indicate 95% confidence intervals. Time points: Postop 1 month (first outpatient visit), 3 months, 6 months, 9 months, and 12 months. The asterisk (*) represents the interaction effect between the reconstruction group and the postoperative time points. Abbreviations: KOQUSS, Korean Quality of Life in Stomach Cancer Patient Study; BI, Billroth I anastomosis; BII, Billroth II anastomosis.

To address potential confounding and selection bias due to the non-randomized study design, IPTW based on propensity scores was applied. The improvement in covariate balance after IPTW is visually summarized in the ASD plot (Figure 1). After weighting, all baseline clinicopathologic variables achieved a high degree of balance; all ASDs were reduced to below the 0.25 threshold recommended by Rubin [12], with most variables achieving an ASD near or below 0.1 (Table 1). Even for variables with relatively higher residual imbalances, such as BMI (ASD 0.20) and tumor location (ASD 0.19), the values remained well within the acceptable limit, confirming that the two groups were sufficiently comparable for longitudinal analysis. After IPTW, no significant differences were observed in baseline characteristics between the two groups, indicating adequate covariate balance (Table 1). In IPTW-adjusted mixed-effects models additionally adjusted for tumor location and histologic differentiation, postoperative QoL was evaluated using the KOQUSS-40 questionnaire and its subscales at five time points: immediately after surgery and at 3, 6, 9, and 12 months (Supplementary Figure S1, Figure 2 and Figure 3). Between-group differences and temporal trends were analyzed using generalized linear mixed models, adjusting for baseline differences in tumor location and histologic differentiation. After adjustment, the total KOQUSS-40 score improved over time in both the BI and BII groups. Although QoL scores were consistently higher in the BII group across all time points, scores in the BI group showed a marked increase after 6 months, and by 12 months postoperatively, the values had converged to levels comparable to those of the BII group. Consequently, a significant main effect for the group was observed, with the BII group maintaining consistently higher total KOQUSS-40 scores than the BI group throughout the study period (*p* = 0.032). However, the interaction between group and time was not significant (*p* = 0.846), indicating that the pattern of recovery over 12 months was similar for both surgical approaches (Figure 2).

Analysis of individual symptom domains revealed several distinct temporal patterns. In particular, the dumping syndrome domain demonstrated a significant group × time interaction (*p* = 0.019) indicating that the trajectory of symptom change differed between the two reconstruction types. Dumping-related symptoms gradually improved over time in the BI group, whereas scores in the BII group remained relatively stable throughout follow-up (Figure 3). Similarly, the constipation domain showed a significant group × time interaction (*p* = 0.012) Constipation scores improved more markedly in the BI group over time, while little change was observed in the BII group (Figure 3).

**Figure 4:**
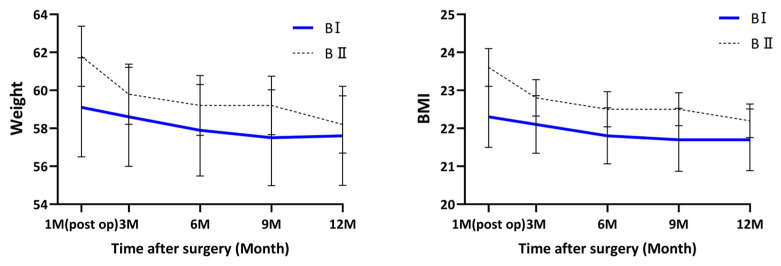
Body weight and BMI change. Data are presented as mean values with 95% confidence intervals. Time points: Postop 1 month (first outpatient visit), 3 months, 6 months, 9 months, and 12 months. Abbreviations: BI, Billroth I anastomosis; BII, Billroth II anastomosis; BMI, Body mass index.

In contrast, other KOQUSS-40 domains—including psychological factors, worry about cancer, general health, bowel habits, and wound-related problems—did not demonstrate significant group × time interactions. Body weight showed a significant time effect (*p* < 0.001), reflecting postoperative weight loss followed by gradual stabilization in both groups. However, no significant group × time interaction was observed for weight, indicating similar temporal patterns of weight change between the BI and BII groups (Figure 4). Among all domains, significant group × time interactions were observed only for dumping (*p* = 0.003), constipation (*p* < 0.001), indicating that these outcomes followed significantly different longitudinal trajectories between the BI and BII groups after IPTW adjustment (Supplementary Table S1, Figure S1).

## 4. Discussion

This analysis evaluated longitudinal QoL outcomes following distal gastrectomy with either BI or BII anastomosis using the KOQUSS-40 instrument. After adjusting for baseline differences in tumor location and histologic differentiation, patients undergoing There was a significant main effect of group (*p* = 0.032), indicating that the BII group maintained higher KOQUSS-40 scores than the BI group throughout the study period. While both groups showed significant improvement over time (time effect, *p* < 0.001), the group-by-time interaction was not statistically significant (*p* = 0.846), suggesting that the recovery trajectories were comparable between the two groups. Although overall QoL scores in the two groups tended to converge over time, the specific domains showing significant changes differed between the BI and BII groups, particularly during the 3- and 6-month postoperative assessments. This divergence suggests that the clinical focus for improving patient QoL should be tailored to the specific reconstruction type and postoperative phase. For instance, clinicians should implement targeted strategies—such as specialized patient education or proactive reassurance regarding expected symptoms—to address the distinct symptomatic profiles observed in each group. Such a personalized approach to postoperative care would be essential for optimizing the quality of life during the critical recovery period.

Regarding indigestion, patients in the BII group consistently reported better scores than those in the BI group; however, no significant differences in changes over time were observed between the groups. A plausible explanation lies in the differing routes of food passage and the size of the remnant stomach. In BI anastomosis, food enters the duodenum directly, perpendicular to the direction of gravity, which may lead to a higher incidence of impaired gastric emptying, especially in the early postoperative phase. This observation aligns with previous reports that endoscopic examinations in BI patients frequently revealed residual food, implying delayed gastric emptying [7]. One potential contributing factor is anastomotic twisting, which has occurred in BI reconstructions [16]. Twisting at the anastomotic site may cause partial mechanical obstruction, further exacerbating food stasis and indigestion symptoms. The anatomical configuration of the BII anastomosis may promote more efficient gastric emptying and reduced stasis due to its wider, gravity-assisted gastrojejunostomy loop. In our analysis, the BII group demonstrated higher scores in the indigestion domain compared with the BI group; however, the difference was not statistically significant, consistent with previous functional evaluations [17]. These observations are further supported by a recent study by Jun et al., which reported that different gastrointestinal reconstruction techniques did not significantly affect postoperative nutritional markers or overall quality of life [18]. Furthermore, a comprehensive meta-analysis by Liu et al. encompassing Billroth I, Billroth II, and Roux-en-Y reconstructions concluded that these techniques offer comparable outcomes regarding long-term functional recovery [19]. Another contributing factor may be that, in BI reconstruction, the remnant stomach is typically larger, whereas the passage through the anastomosis may be relatively narrow, predisposing patients to food stasis. This anatomical configuration may contribute to persistent symptoms of indigestion observed in BI patients. Furthermore, BII reconstruction bypasses the duodenum, potentially reducing biliary reflux. Although reflux was not a significant factor in our cohort, studies have demonstrated that reflux symptoms measured by KOQUSS-40 do not always correlate with endoscopic findings. Therefore, symptom-based QoL tools may capture clinically relevant discomfort that traditional diagnostic methods might overlook.

Notably, the dumping syndrome domain showed a significant group-by-time interaction (*p* < 0.05), suggesting distinct temporal recovery patterns between the two groups. In the BI group, dumping scores improved from 81.0 at 6 months to 83.4 at 12 months, whereas the BII group exhibited relative stability. While BII reconstruction has traditionally been considered more prone to dumping due to rapid gastric emptying [20,21], our findings suggest that the clinical manifestation of dumping-like symptoms may be influenced by a more complex interplay of factors. For instance, hormonal responses such as GLP-1 secretion and insulin release have been proposed as underlying mechanisms, though their relative impacts likely vary by individual physiological adaptation [22,23]. Furthermore, early postoperative discomfort in the BI group—potentially attributable to transient delayed gastric emptying from anastomotic tension—may have overlapped with symptoms captured in the dumping domain. The gradual improvement in the BI group might therefore reflect the resolution of these functional disturbances rather than a direct reduction in dumping severity. As this study was exploratory, further research using diagnostic tools capable of differentiating stasis-related discomfort from true dumping syndrome is warranted to validate these observations.

Similarly, a significant group-by-time interaction was identified for constipation. Constipation outcomes improved more markedly over time in the BI group, whereas no significant changes were observed in the BII group during follow-up. This pattern differs from the KOQUSS multicenter study, in which the BII group demonstrated a more pronounced change in scores over time compared with the BI group [24]. However, our findings align with the study by Pomortsev et al., which demonstrated that the BI anastomosis group had higher constipation symptom scores than the Roux-en-Y group, based on EORTC QLQ-C30 analysis [25]. Furthermore, although a 2023 study comparing BI and BII reconstructions using the EORTC QLQ-C30 found no significant difference in the constipation domain between the two groups, our study—utilizing the KOQUSS-40 questionnaire—revealed a distinct symptomatic divergence [7]. These finding suggests that the KOQUSS-40 may be more sensitive for detecting subtle longitudinal changes in bowel habits within the Korean gastrectomy population. Our findings indicate that BI-related constipation is more transient and reversible. One possible explanation is that BI reconstruction generally preserves a larger gastric remnant than BII reconstruction, which may predispose patients to delay intestinal transit through early postoperative duodenal passage [17,26]. However, compensatory mechanisms gradually restore bowel motility. This restoration may be facilitated by the physiologic gastroduodenostomy created during BI reconstruction, which preserves duodenum continuity and maintains more physiologic neurohormonal signaling involved in gastrointestinal motility [17,27].

This study has some limitations. First, as a retrospective, single-center study, this analysis is subject to potential selection bias and confounding by indication. Although IPTW was applied to adjust for baseline imbalances, the retrospective nature of the study remains an inherent limitation. Furthermore, because the reconstruction method was determined at the surgeon’s discretion, unmeasured clinical factors—such as intraoperative anatomical constraints or subtle clinical judgments—may have influenced both the choice of procedure and subsequent QoL outcomes. While IPTW balances measured covariates, residual confounding from these unobserved factors cannot be entirely excluded. Therefore, causal interpretations should be made with caution, and our findings should be considered associative rather than definitive. Second, although the BII group maintained a higher BMI, which could potentially favor their QoL scores, previous research suggests that QoL is more strongly influenced by the occurrence of weight loss itself rather than by the absolute BMI reached [28]. Given that both groups showed declining BMI trends yet largely remained within the normal range, the observed QoL differences are unlikely to be solely attributable to BMI variation. Third, the absence of preoperative baseline QoL data limits our ability to evaluate the true magnitude of postoperative changes from a pre-surgical state. Although the KOQUSS-40 is specifically designed for postoperative symptom assessment, the lack of a baseline may complicate the interpretation of early longitudinal trends. Since routine QoL assessments were not performed prior to surgery during the study period, no structured preoperative symptom data were available. To address this, we defined the first postoperative visit as the initial reference point in our longitudinal analysis and employed GLMM to adjust for clinicopathologic variables, thereby minimizing the potential impact of baseline imbalances on the observed recovery trajectories. Despite these limitations, the findings provide valuable longitudinal insights; however, future multicenter studies with balanced cohorts and extended follow-up are warranted. Overall, while the reconstruction type was associated with differences in longitudinal QoL outcomes, the observed effects were domain-specific and modest in magnitude. Fourth, multiple comparisons were performed across several QoL domains without formal adjustment for multiplicity. Therefore, these domain-specific findings should be interpreted with caution and considered exploratory rather than confirmatory. Despite these modest differences, the distinct recovery trajectories identified in specific symptoms, such as dumping and constipation, provide valuable insights for personalized postoperative care. These findings highlight the importance of interpreting QoL outcomes within a broader clinical context rather than relying solely on statistical comparisons. Therefore, while our results should be interpreted with caution regarding their clinical relevance relative to the Minimal Clinically Important Difference (MCID), they offer a meaningful basis for informing patients about expected functional recovery patterns after gastrectomy.

## 5. Conclusions

In conclusion, this study demonstrates that the Billroth II (BII) reconstruction provides modestly higher overall quality of life (QoL) throughout the first postoperative year compared to Billroth I (BI). Although recovery trajectories for total scores were comparable, the use of the KOQUSS-40 identified clinically meaningful differences in specific symptom domains. Specifically, while the BI group experienced higher early postoperative distress in dumping and constipation domains, they showed significant longitudinal improvement, whereas the BII group remained stable. These findings, robust after IPTW adjustment, suggest that early symptomatic variations tend to attenuate and converge within one year. Therefore, while BII may be associated with better overall symptomatic stability, the distinct recovery patterns observed in the BI group provide a crucial basis for tailored patient counseling. Our results underscore the necessity of domain-specific, longitudinal assessments to optimize personalized postoperative care strategies after distal gastrectomy.

## Figures and Tables

**Table 1:** Baseline clinicopathological characteristics of the patients.

		BI (*n* = 51)	BII (*n* = 183)	*p*-Value	ASD	IPTW *p*-Value	IPTW ASD
Age		63.06 ± 10.49	65.63 ± 10.08	0.149	0.25	0.067	0.10
Sex	Male	30 (58.8)	108 (59.0)	0.980	0.00	0.691	0.04
	Female	21 (41.2)	75 (41.0)				
BMI (kg/m^2^)		23.29 ± 3.20	24.45 ± 3.55	0.059	0.34	0.233	0.20
ASA	1	5 (9.8)	22 (12.0)	0.413	0.07	0.268	0.04
	2	36 (70.6)	129 (70.5)		0.00		0.11
	3	9 (17.6)	32 (17.5)		0.00		0.12
	4	1 (2.0)	0 (0.0)		0.20		0.12
Tumor Location	Upper-middle	0 (0.0)	4 (2.2)	0.033	021	0.183	0.19
	Middle	3 (5.9)	35 (19.1)		0.41		0.00
	Lower	48 (94.1)	144 (78.7)		0.46		0.05
Tumor Size (cm)		2.38 ± 1.24	2.54 ± 1.18	0.246	0.13	0.771	0.08
pStage	I	44 (86.3)	153 (83.6)	0.925	0.08	0.409	0.03
	II	6 (11.8)	26 (14.2)		0.07		0.09
	III	1 (2.0)	4 (2.2)		0.02		0.10
Differentiation	Differentiated	18 (35.3)	100 (54.6)	0.024	0.04	1.000	0.00
	Undifferentiated	32 (62.7)	76 (41.5)		0.44		0.00
	Other	1 (2.0)	7 (3.8)		0.11		0.01
Postop cTx		7 (13.7)	24 (13.1)	0.909	0.02	0.565	0.06

Abbreviations: BI, Billroth I anastomosis; BII, Billroth II anastomosis; ASD: absolute standardized difference; IPTW: inverse probability of treatment weighting; BMI: body mass index; ASA: American Society of Anesthesiologists; pStage: pathologic stage; Postop cTx: postoperative chemotherapy.

## Data Availability

The data presented in this study are available on request from the corresponding author. The data are not publicly available due to privacy and ethical restrictions.

## Supplementary Materials

The following supporting information can be downloaded at: https://www.mdpi.com/article/10.3390/jcm15103738/s1, Table S1: The results of time-course variables from analyses using a generalized linear mixed model; Figure S1: The results of IPTW-adjusted mixed-effects models additionally adjusted for tumor location and histologic differentiation.

## Abbreviations

The following abbreviations are used in this manuscript:

BI	Billroth I
BII	Billroth II
BMI	Body Mass Index
KOQUSS-40	Korean Quality of Life Questionnaire for Gastric Cancer Surgery (40 items)
