# Supplementary material for: Longitudinal Quality-of-Life Trajectories Following Laparoscopic Distal Gastrectomy: A Comparison Between Billroth I and II Reconstruction Using the KOQUSS-40 Questionnaire
Source: J Clin Med. 2026 May 13;15(10):3738. doi: 10.3390/jcm15103738 (PMC13207270; doi:10.3390/jcm15103738)
Supplement: Supplementary file 1 [file jcm-15-03738-s001.zip › jcm-4237405-supplementary.pdf]

Supplementary Table S1. The results of time-course variables from analyses using a generalized linear mixed model

Variable	Group		p	Adjusted p[3]	Source	LMM		IPTW-adjusted LMM	
	BⅠ (n=51, 21.8%)	BⅡ (n=183, 78.2%)				Unadjusted p[5]	Adjusted p[6]	Unadjusted p[7]	Adjusted p[8]
**Indigestion**									
1 month (post op)	43.9±23.7[a]	49.7±19.4[a]	.236[2]	.354	Group	.011	.009	<.001	<.001
3 months	61.4±20.1[b]	66.1±18.0[b]	.228[2]	.240	Time	<.001	<.001	<.001	<.001
6 months	63.9±16.9[b]	70.2±18.0[c]	.024[2]	.031	Group ×Time	.964	.991	.656	.692
9 months	63.9±18.0[b]	73.5±16.3[c]	.001[2]	.002					
12 months	68.0±16.4[b]	72.2±17.7[c]	.127[2]	.108					
**p[4]**	<.001	<.001							
**Dysphagia**									
1 month (post op)	86.1±14.2	82.7±20.7	.710[2]	.868	Group	.329	.409	.055	.073
3 months	80.3±25.0	86.4±16.0	.518[2]	.538	Time	.161	.168	.001	.001
6 months	80.1±19.3	86.0±18.8	.035[2]	.040	Group ×Time	.827	.812	.875	.860
9 months	84.3±17.8	86.8±15.5	.395[2]	.445					
12 months	86.9±15.6	86.0±18.4	.923[2]	.940					
**p[4]**	.080	.068							
**Reflux**									
1 month (post op)	89.1±13.8	86.5±19.4	.656[2]	.539	Group	.943	.990	.589	.497

3 months	86.6±18.8	84.9±17.7	.450[2]	.689	Time	.382	.395	.135	.140
6 months	85.6±16.8	85.6±17.2	.949[2]	.943	Group ×Time	.947	.932	.962	.968
9 months	89.1±14.9	87.1±13.8	.221[2]	.254					
12 months	86.2±15.0	83.7±19.0	.640[2]	.850					
**p[4]**	.644	.141							
**Dumping**									
1 month (post op)	87.2±11.9 [a]	86.5±13.2	.745[2]	.697	Group	.906	.598	.659	.291
3 months	87.0±11.4 [ab]	88.4±11.7	.319[2]	.405	Time	.063	.051	.046	.041
6 months	81.2±17.8 [b]	89.2±12.2	<.001[2]	<.001	Group ×Time	.027	.019	.006	.003
9 months	82.2±15.3 [ab]	88.4±12.9	.005[2]	.009					
12 months	83.4±14.1 [ab]	86.8±15.4	.062[2]	.060					
**p[4]**	.006	.078							
**Bowel habit**									
1 month (post op)	82.8±12.4 [a]	78.9±17.1	.348[2]	.383	Group	.813	.622	.899	.607
3 months	75.8±19.3 [ab]	77.2±16.7	.828[2]	.873	Time	.146	.132	.079	.072
6 months	72.9±18.9 [b]	76.9±18.7	.152[2]	.114	Group ×Time	.335	.292	.296	.223
9 months	73.3±15.1 [b]	78.0±16.7	.045[2]	.038					
12 months	79.4±13.8 [ab]	77.5±17.3	.771[2]	.812					
**p[4]**	.003	.734							
**Constipation**									

1 month (post op)	76.1±29.5 [a]	83.7±22.2	.138[2]	.273	Group	.014	.025	.002	.004
3 months	86.4±17.3 [ab]	86.5±19.0	.764[2]	.758	Time	<.001	<.001	<.001	<.001
6 months	87.2±20.1 [b]	87.6±18.1	.986[2]	.779	Group ×Time	.014	.015	<.001	<.001
9 months	89.9±15.1 [b]	86.9±17.1	.318[2]	.293					
12 months	89.3±15.1 [b]	87.3±18.4	.653[2]	.802					
**p[4]**	.001	.197							
**Psychological factor**									
1 month (post op)	78.1±17.9	82.0±16.1	.227[2]	.255	Group	.016	.031	.021	.057
3 months	79.1±20.1	83.4±17.0	.184[2]	.281	Time	.250	.231	.006	.006
6 months	73.2±20.3	82.1±17.0	.005[2]	.007	Group ×Time	.645	.582	.083	.059
9 months	73.0±20.2	83.3±16.7	<.001[2]	<.001					
12 months	78.1±15.7	79.8±19.1	.264[2]	.252					
**p[4]**	.088	.060							
**General health**									
1 month (post op)	69.3±18.7	70.9±18.5[a]	.708[2]	.842	Group	.301	.299	.261	.555
3 months	75.5±19.6	75.5±19.5[b]	.938[2]	.736	Time	.037	.034	<.001	<.001
6 months	74.3±19.2	74.8±20.9[b]	.708[2]	.620	Group ×Time	.894	.888	.696	.639
9 months	72.2±18.4	75.9±19.0[b]	.168[2]	.184					
12 months	74.3±18.6	74.8±20.3[b]	.997[2]	.988					
**p[4]**	.225	.006							

**Wound related problem**									
1 month (post op)	79.2±15.8[a]	76.4±14.7[a]	.121[2]	.085	Group	.380	.199	.006	<.001
3 months	91.9±10.3[b]	89.9±12.2[b]	.402[2]	.447	Time	<.001	<.001	<.001	<.001
6 months	88.2±15.1[b]	90.3±12.0[b]	.579[2]	.764	Group ×Time	.430	.399	.073	.052
9 months	90.6±11.0[b]	90.4±11.2[b]	.955[2]	.826					
12 months	91.1±12.4[b]	90.4±12.1[b]	.640[2]	.564					
**p[4]**	<.001	<.001							
**KOQUSS40**									
1 month (post op)	75.6±10.8[a]	77.7±12.3[a]	.123[2]	.204	Group	.016	.036	<.001	.004
3 months	78.5±11.3[ab]	81.1±11.1[b]	.166[2]	.212	Time	<.001	<.001	<.001	<.001
6 months	76.8±12.2[ab]	81.5±12.3[b]	.011[2]	.018	Group ×Time	.821	.877	.573	.682
9 months	78.3±10.9[ab]	82.5±11.2[b]	.017[2]	.027					
12 months	80.7±9.7[b]	81.2±13.0[b]	.331[2]	.307					
**p[4]**	.043	<.001							

Abbreviations: BI, Billroth Ⅰ anastomosis; BII, Billroth Ⅱ anastomosis; LMM, Linear mixed-effects models; IPTW, Inverse probability of treatment weighting; post op, KOQUSS, Korean Quality of Life Questionnaire for Gastric Cancer Surgery

[1] P values were derived from independent t-test.

[2] P values were derived from Mann-Whitney's U test.

[3] P values were derived by analysis of covariance (ANCOVA) or ranked ANCOVA adjusted for tumor location, and differentiation which were significant in Table 1.

[4] P values were derived by repeated measured ANOVA or Friedman's test. Means with different superscript letters are significantly different ($p < 0.05$).

[5] P values were derived from generalized linear mixed-effects models (GLMMs). The model included fixed effects for group, time (1, 3, 6, 9, and 12 months), and the group × time interaction, with a random intercept for each participant and an

unstructured covariance matrix for repeated measures. All available data were included in the analysis using linear mixed-effects models with restricted maximum likelihood estimation, under the assumption that missing data were missing at random (MAR).

[6] P values were derived from GLMMs adjusted for tumor location and histologic differentiation, which were significant in Table 1.

[7] P values were derived from IPTW-adjusted mixed-effects models.

[8] P values were derived from IPTW-adjusted mixed-effects models including tumor location and histologic differentiation as covariates.

Shapiro-Wilk’s test was employed for test of normality assumption.

Supplementary Figure S1. The results of IPTW-adjusted mixed-effects models additionally adjusted for tumor location and histologic differentiation.

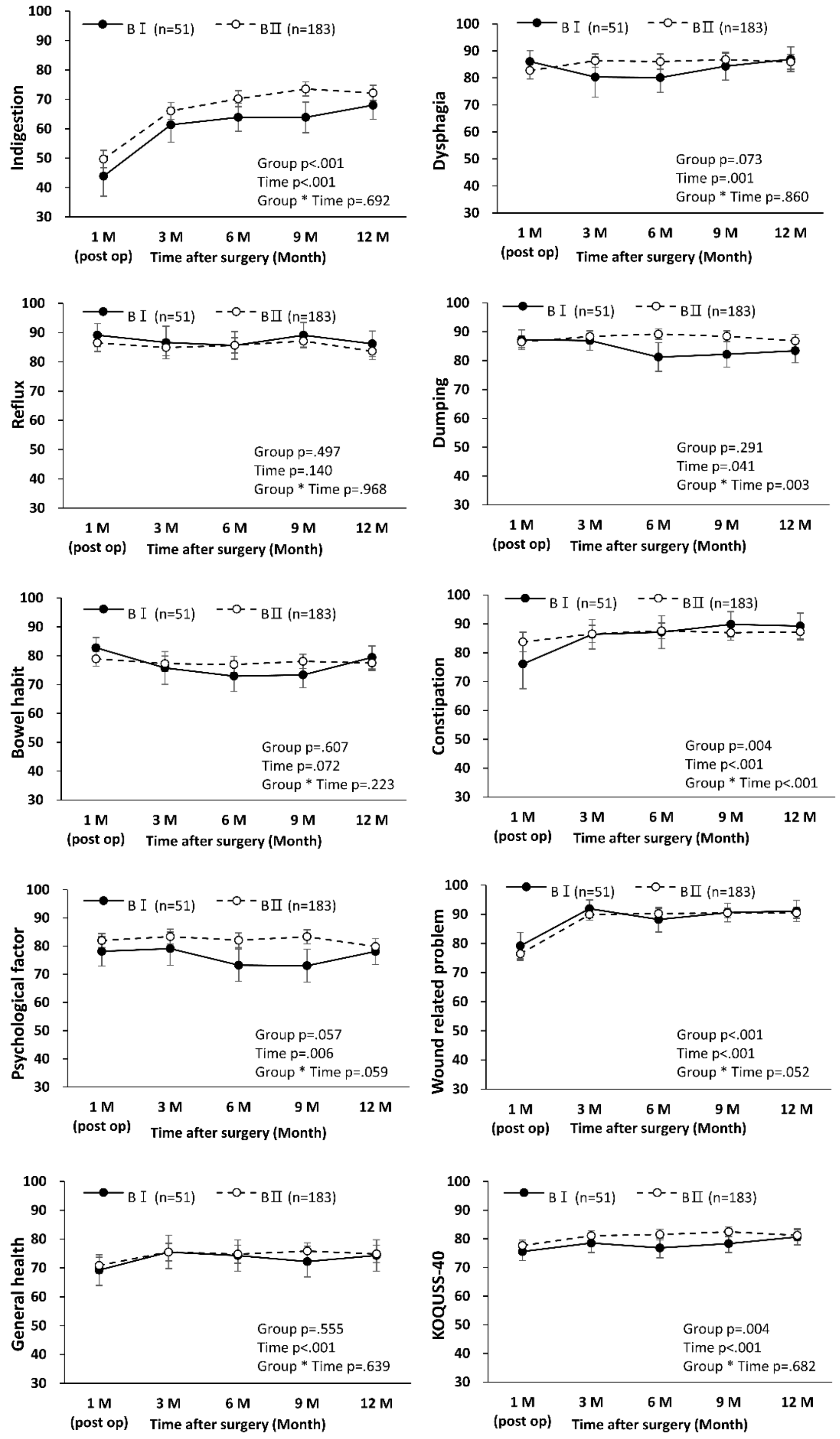

Abbreviations: BI, Billroth Ⅰ anastomosis; BII, BillrothⅡanastomosis; post op, post operation; KOQUSS, Korean Quality of Life Questionnaire for Gastric Cancer Surgery
